# Temporal Dynamics of the Adult Female Lower Urinary Tract Microbiota

**DOI:** 10.1128/mBio.00475-20

**Published:** 2020-04-21

**Authors:** Travis K. Price, Birte Wolff, Thomas Halverson, Roberto Limeira, Linda Brubaker, Qunfeng Dong, Elizabeth R. Mueller, Alan J. Wolfe

**Affiliations:** a Department of Microbiology and Immunology, Stritch School of Medicine, Loyola University Chicago, Maywood, Illinois, USA; b Department of Obstetrics & Gynecology, Loyola University Medical Center, Maywood, Illinois, USA; c Department of Urology, Loyola University Medical Center, Maywood, Illinois, USA; d Department of Obstetrics, Gynecology and Reproductive Sciences, Division of Female Pelvic Medicine and Reconstructive Surgery, University of California San Diego, La Jolla, California, USA; e Department of Medicine, Stritch School of Medicine, Loyola University Chicago, Maywood, Illinois, USA; University of Hawaii at Manoa

**Keywords:** lower urinary tract, menstruation, metaculturomics, microbiome, sex, urinary microbiota, urobiome, 16S rRNA gene sequencing

## Abstract

Temporal dynamics of certain human microbiotas have been described in longitudinal studies; variability often relates to modifiable factors or behaviors. Early studies of the urinary microbiota preferentially used samples obtained by transurethral catheterization to minimize vulvovaginal microbial contributions. Whereas voided specimens are preferred for longitudinal studies, the few studies that reported longitudinal data were limited to women with lower urinary tract (LUT) symptoms, due to ease of accessing a clinical population for sampling and the impracticality and risk of collecting repeated catheterized urine specimens in a nonclinical population. Here, we studied the microbiota of the LUT of nonsymptomatic, premenopausal women using midstream voided urine (MSU) specimens to investigate relationships between microbial dynamics and personal factors. Using 16S rRNA gene sequencing and a metaculturomics method called expanded quantitative urine culture (EQUC), we characterized the microbiotas of MSU and periurethral swab specimens collected daily for approximately 3 months from a small cohort of adult women. Participants were screened for eligibility, including the ability to self-collect paired urogenital specimens prior to enrollment. In this population, we found that measures of microbial dynamics related to specific participant-reported factors, particularly menstruation and vaginal intercourse. Further investigation of the trends revealed differences in the composition and diversity of LUT microbiotas within and across participants. These data, in combination with previous studies showing relationships between the LUT microbiota and LUT symptoms, suggest that personal factors relating to the genitourinary system may be an important consideration in the etiology, prevention, and/or treatment of LUT disorders.

## Abstract (precis)

Following the discovery of the collective human urinary microbiota, important knowledge gaps remain, including the stability and variability of this microbial niche over time. Initial urinary studies preferentially utilized samples obtained by transurethral catheterization to minimize contributions from vulvovaginal microbes. However, catheterization has the potential to alter the urinary microbiota; therefore, voided specimens are preferred for longitudinal studies. In this report, we describe microbial findings obtained by daily assessment over 3 months in a small cohort of adult women. We found that, similarly to vaginal microbiotas, lower urinary tract (LUT) microbiotas are dynamic, with changes relating to several factors, particularly menstruation and vaginal intercourse. Our study results show that LUT microbiotas are both dynamic and resilient. They also offer novel opportunities to target LUT microbiotas by preventative or therapeutic means, through risk and/or protective factor modification.

## INTRODUCTION

The microorganisms of our bodies are collectively known as the human microbiota (1). These communities of bacteria can influence many aspects of health and disease. The balance of benefit versus harm depends largely on the overall state of the microbiota in terms of distribution, diversity, and composition (2). Understanding the variables associated with microbial changes is an important first step to intentional modulation of the microbiota. Compared to higher-biomass microbial niches, such as the gut, little is known about the temporal dynamics of the female urinary microbiota.

Temporal changes in the vaginal microbiota have been described previously. Overall, the vaginal microbiota shows low microbial constancy and high species turnover over time, but the dynamics vary widely among individuals (3, 4). Vaginal microbiotas can also be resilient (5), meaning that they return to a baseline state following disruption. Alterations in vaginal microbiotas relate to vaginal health (e.g., bacterial vaginosis [BV]) (5, 6) and personal factors (e.g., menstruation, sexual intercourse, contraception, pregnancy, menopausal status) (3, 5, 7–14). The incidence of BV can also fluctuate (15) and relates to personal factors (16–21). Altogether, these data suggest possible interplay among personal factors and practices, microbial dysbioses, and vaginal health.

Likewise, urinary tract infection (UTI) risk in women is associated with similar personal factors (22–26). These data, in combination with the discovery of resident microbiotas in the bladders of women (27–32), provide evidence for a proposal of similar interplay for the female lower urinary tract (LUT), consisting of the bladder and urethra, which are in close proximity to the vagina. To date, only one published study assessed the urinary microbiota longitudinally. In males, Nelson et al. showed that voided urine specimens collected at 1-month intervals were significantly more similar within a participant than between participants (33), suggesting microbial stability. They found that some bacterial taxa (e.g., *Propionibacterium* and *Lactobacillus*) had long durations of colonization, while others (e.g., *Corynebacterium*, *Anaerococcus*, *Staphylococcus*, and *Prevotella*) were more infrequent (33). Furthermore, BV-associated taxa (e.g., *Mycoplasma*, *Ureaplasma*, and *Sneathia*) were detected only in sexually experienced males (33). These data show that urinary microbiotas in males are dynamic and may relate to personal factors. Whether this is true for the female LUT microbiota and whether daily assessment of the microbiota would provide further clarity remain unclear and serve as the primary objectives of this study.

In this study, we characterized the microbiotas of midstream voided urine (MSU) and periurethral swab samples in specimens collected daily for 3 months from premenopausal adult women without LUT symptoms. This report represents a secondary analysis of a previously described study (34), where we measured the change in the ratio of urinary pathogens and *Lactobacillus* spp. within the LUT in response to oral probiotic use. In this double-blind randomized controlled trial, we found no effect of oral probiotic use on the LUT microbiota (34). Here, our aims were to describe the longitudinal microbiotas of MSU and periurethral swab specimens in this participant population and to determine whether correlations existed between temporal changes in the microbiotas and participant-reported biological and behavioral factors.

We found that, similarly to vaginal microbiota, LUT microbiota are dynamic and are associated with specific biological and behavioral factors, particularly menstruation and vaginal intercourse. Our report provides the first descriptive analysis of temporal changes in the LUT microbiota. It shows that these microbiota are relatively stable during health. They can be modulated temporarily by certain behavioral and biological factors. However, the LUT microbiota of young healthy women are remarkably resilient. It also offers novel opportunities to target the LUT microbiota for preventative or therapeutic means, through lifestyle modifications.

## RESULTS

### Study design and patient demographics.

Because posturethral (e.g., vulval and/or vaginal) microbes are often present in MSU samples (35, 36), it was essential to include a control for specimen quality. Thus, we used a periurethral swab to measure specimen collection compliance. We prescreened participants, seeking those whose periurethral swab microbiota and MSU microbiota differed substantially, allowing us to derive conclusions that exclusively relate to the LUT microbiota rather than to those of the periurethral area.

We screened 12 participants for eligibility by calculating Bray-Curtis dissimilarity index values between microbiotas of paired MSU and periurethral specimens collected over 3 consecutive days. The Bray-Curtis dissimilarity index is used to quantify the compositional dissimilarity between two sites. This index produces values between 0 and 1, with a value of 0 assigned to completely similar specimens. Four participants did not meet eligibility criteria (see Table S1 in the supplemental material).

10.1128/mBio.00475-20.3 TABLE S1 Bray-Curtis dissimilarity values for paired specimens during the screening phase. Download Table S1, PDF file, 0.1 MB. Copyright © 2020 Price et al. 2020 Price et al. This content is distributed under the terms of the Creative Commons Attribution 4.0 International license.

**FIG 1:**
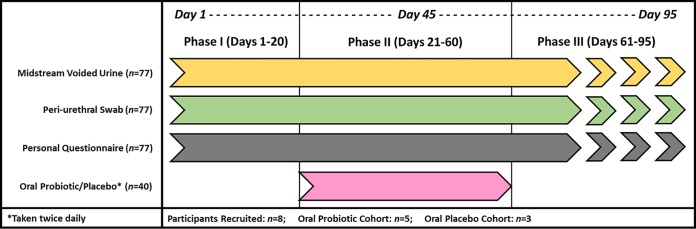
ProFUM clinical study design outline. Midstream voided urine (MSU) and periurethral swab specimens were self-collected daily from 8 participants from day 1 to day 74, followed by weekly collection through day 95. Daily questionnaires were completed on each of these days. The study design was divided into three phases: phase I (days 1 to 20), phase II (days 21 to 60), and phase III (days 61 to 95). During phase II, the participants were randomized (2:1) to take an oral probiotic or placebo twice daily. This assignment was performed in a double-blind manner.

The other 8 participants entered into the study, which had 3 phases (Fig. 1). During phase I (days 1 to 20) and phase II (days 21 to 60), all participants collected daily MSU and periurethral specimens and completed a questionnaire. During phase II, participants were randomized to take an oral probiotic or placebo. The details of this part of the study have been published previously (34). During phase III (days 61 to 95), all participants collected MSU and periurethral specimens and completed the questionnaire on a daily basis from day 61 to day 74, followed by weekly collection and questionnaire completion from day 74 to day 95. Specimens were delivered each morning for laboratory analysis.

None of the participants had symptoms of urinary incontinence or a history of gynecological surgery, kidney stones, or recurrent urinary tract infections (Table 1).

**TABLE 1:** Demographics and clinical characteristics of the participants^*a*^

ProFUM participant demographic	Values
Age (yrs), mean (SD)	29 (±5)
	
Race/ethnicity	
White/Caucasian	5 (62.5)
Asian	2 (25)
Black/African-American	1 (12.5)
	
BMI, mean (SD)	24.6 (±6.2)
	
Prior pregnancy	1 (12.5)
Vaginal delivery	1 (12.5)
	
Menstrual cycle	
Regular (every 20–40 days)	5 (62.5)
Irregular	3 (37.5)
None	0 (0)
	
Menstrual hygiene product use	
Tampon	5 (62.5)
Pad	4 (50)
Menstrual cup	2 (25)
None	0 (0)
	
Sexually active	
Frequency of sexual activity	8 (100)
Daily	1 (12.5)
Wkly	2 (25)
Monthly	3 (37.5)
Not in past yr	2 (25)
Type of sexual activity (in past yr) (*n* = 6)	
Vaginal (penetrative with penis)	6 (100)
Vaginal (penetrative with toy/fingers)	6 (100)
External stimulation	2 (33.3)
Cunnilingus (receiving)	6 (100)
Anal (penetrative)	0 (0)
Current sexual partners	
One male partner	6 (75)
Multiple male partners	1 (12.5)
No current partners	1 (12.5)
Method of birth control	
Condom use	2 (25)
Intrauterine device (IUD)	4 (50)
Oral contraceptive	2 (25)
	
Hygiene	
No. of showers (wkly), mean (SD)	6 (±1)
No. of baths (wkly), mean (SD)	1 (±1)
	
No. of bowel movements (wkly), mean (SD)	8 (±3)
	
Diet	
Follows a special diet	1 (12.5)
Alcohol consumption	8 (100)

a Data represent number (percent) of participants (*n* = 8) unless otherwise indicated. BMI, body mass index.

### Microbiota characteristics.

**FIG 2:**
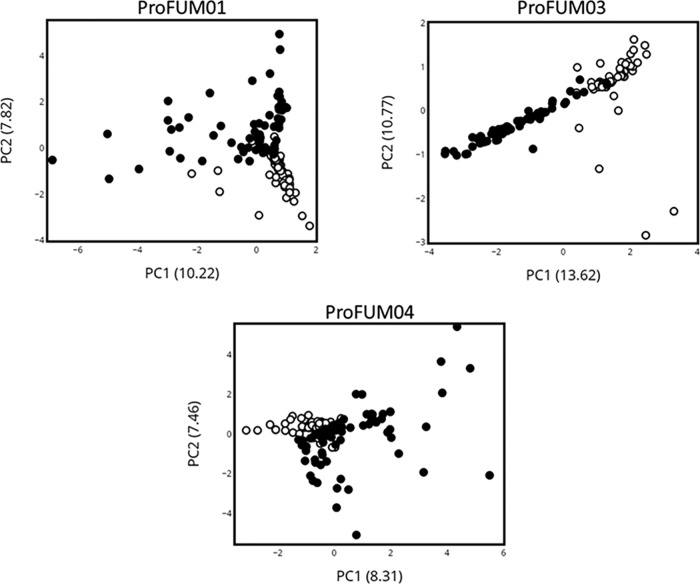
Principal-coordinate analyses of microbiota of specimens from participants ProFUM01, ProFUM03, and ProFUM04. Analysis was done using MSU (closed circles) and periurethral (open circles) microbiota data collected from three participants indicated as follows: top left, ProFUM01; top right, ProFUM03; bottom, ProFUM04. Graphs plot the first and second principal coordinates of the data. Percentages of total variance explained by each principal coordinate are shown in parentheses.

To evaluate if the MSU and periurethral microbiotas remained distinct in the participants over the course of the study, we compared their compositions using the Bray-Curtis dissimilarity index. The microbiotas of the paired (i.e., collected on same day) MSU and periurethral swab specimens remained distinct (Table S2). Similarly, principal-coordinate analysis (PCoA) results showed that, in general, the microbiotas of the MSU and periurethral area in the individual participants remained distinct over the course of the 90-day study (Fig. 2).

10.1128/mBio.00475-20.4 TABLE S2 Overview of Bray-Curtis dissimilarity values comparing MSU and periurethral microbiota of the ProFUM participants on the basis of EQUC data. Download Table S2, PDF file, 0.05 MB. Copyright © 2020 Price et al. 2020 Price et al. This content is distributed under the terms of the Creative Commons Attribution 4.0 International license.

### Qualitative and quantitative description of the longitudinal LUT microbiota and microbiome.

**FIG 3:**
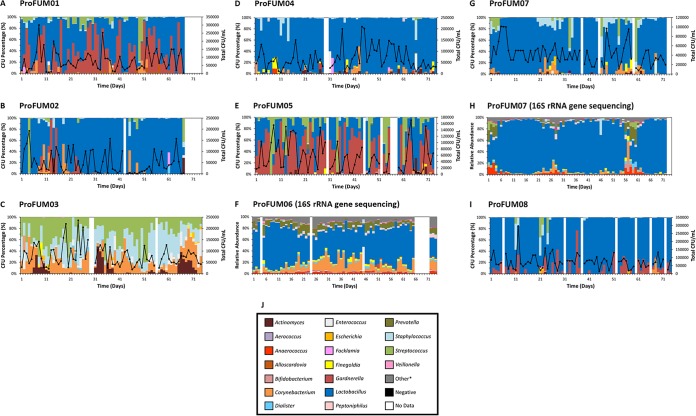
Microbiota profiles of MSU specimens from all ProFUM participants. Microbiota profiles are shown as stacked bar graphs depicting the relative abundances (*y* axes) of various genera from MSU specimens over time in chronological order (*x* axes). Bars that appear white refer to days where no specimen was collected, received, or stored. A legend containing the most common genera is shown in panel J. “Other” refers to the combined relative abundances for all taxa not included in the 20 most abundant taxa. Data were generated using modified EQUC (panels A, B, C, D, E, G, and I) or 16S rRNA gene sequencing (panels F and H). *Other, used only for panels F and H.

We evaluated the composition of the participants’ LUT microbiotas using a modified version of the expanded quantitative urine culture (EQUC) protocol. In parallel, the microbiotas of two participants were evaluated using 16S sequencing (Fig. 3). In particular, the data for participant ProFUM7 show that EQUC and 16S sequencing provided similar results.

Three general patterns were observed: (i) *Lactobacillus* predominance (ProFUM02, ProFUM04, ProFUM07, ProFUM08), (ii) alternating *Lactobacillus* and *Gardnerella* predominance (ProFUM01, ProFUM05), and (iii) changing ratios of *Streptococcus*, *Staphylococcus*, and *Corynebacterium* (ProFUM03). *Lactobacillus* predominance was found to be restricted to a single species (ProFUM07) or alternating species (ProFUM 02, ProFUM04, ProFUM08). Less-prevalent taxa were observed in each participant; some were associated with personal factors (see below). CFU counts per milliliter ranged across 5 orders of magnitude, with a maximum of approximately 250,000. A detailed description of each participant and their MSU composition is presented (Table S3).

10.1128/mBio.00475-20.5 TABLE S3 Qualitative description of the microbial temporal dynamics of MSU specimens of the ProFUM participants represented in Fig. 3. Download Table S3, PDF file, 0.1 MB. Copyright © 2020 Price et al. 2020 Price et al. This content is distributed under the terms of the Creative Commons Attribution 4.0 International license.

To quantify the stability for both the microbiotas and microbiomes of the MSU and periurethral specimens, we used the Jensen-Shannon divergence (JSD) statistic, representing a method of measuring the similarity between two probability distributions. Median JSD values were lower for MSU specimens than for periurethral specimens (see Fig. S1A in the supplemental material), showing that the periurethral microbiota is more variable within an individual than is the MSU microbiota. However, the range of JSD values for the MSU microbiotas was large, indicating that periods of heightened variability exist (Fig. S1B).

10.1128/mBio.00475-20.1 FIG S1 Distribution of Jensen-Shannon divergence values for MSU and periurethral microbiota from all ProFUM participants. Box plots depict the range of JSD values of microbiota data (EQUC of ProFUM, 1 to 5, 7, and 8) and microbiome data (16S rRNA gene sequencing of ProFUM06 and ProFUM07) corresponding to MSU specimens (A) and periurethral specimens (B) from all applicable ProFUM participants. Median scores are depicted by the solid lines in each box. The distributions of raw values are depicted by the overlaid circles. Download FIG S1, TIF file, 0.2 MB. Copyright © 2020 Price et al. 2020 Price et al. This content is distributed under the terms of the Creative Commons Attribution 4.0 International license.

### Menstruation and LUT microbiota variability.

Menstruation was reported by all eight participants. Three participants reported having irregular menstrual cycles, while five reported having regular cycles defined as one occurring every 20 to 40 days (Table 1). Five participants reported using a form of contraception/birth control other than condom use (Table 1). JSD values were higher for the MSU microbiotas during menstruation for most participants (Table 2). In contrast, JSD values for the periurethral microbiotas were not significantly higher during menstruation, except for participant ProFUM02 (*P < *0.001) (Table 2). These data show that a significant association exists between menstruation and variable MSU microbiotas but not between menstruation and periurethral microbiotas.

**TABLE 2:** Association between Jensen-Shannon divergence values for MSU and periurethral microbiota and participant-reported menstruation

Participant	Median JSD value or *P* value^*a*^					
	MSU microbiota			Periurethral microbiota		
	Yes (*n*)	No (*n*)	*P* value	Yes (*n*)	No (*n*)	*P* value
ProFUM01	0.200 (15)	0.145 (52)	0.109	0.355 (15)	0.324 (50)	0.729
ProFUM02	0.316 (7)	0.127 (59)	**0.039**	0.377 (7)	0.172 (55)	**<0.001**
ProFUM03	0.137 (18)	0.100 (53)	**0.041**	0.127 (16)	0.112 (47)	0.313
ProFUM04	0.122 (12)	0.059 (60)	**0.023**	0.320 (10)	0.253 (56)	0.088
ProFUM05	0.101 (3)	0.129 (64)	0.989	0.240 (3)	0.252 (57)	0.873
ProFUM06 (16S)	0.094 (7)	0.072 (59)	0.540			
ProFUM07	0.078 (41)	0.052 (28)	**0.014**	0.246 (41)	0.322 (28)	0.095
ProFUM07 (16S)	0.070 (43)	0.048 (29)	**<0.001**			
ProFUM08	0.098 (19)	0.068 (48)	**0.047**	0.227 (19)	0.297 (47)	0.051

a Median JSD values are shown for MSU and periurethral microbiota on days when the participant reported (“Yes”) or did not report (“No”) menstruation. The Mann-Whitney U test was used for statistical analyses. *P* values of <0.05 represent statistical significance (bold). *n*, number of participants.

To determine the nature of MSU microbiota variability associated with menstruation, we assayed for differences in microbial composition and diversity. Median alpha-diversity measures during menstruation were significantly higher for most participants (Table S4), particularly for those participants who had previously showed high MSU microbiota variability during menstruation (Table 2). We also observed numerous differences in the frequencies of detected taxa during menstruation. Table S5A lists the bacterial taxa that showed significantly different frequencies of detection between menstruation and nonmenstruation days. Five participants (ProFUM01, ProFUM02, ProFUM03, ProFUM04, and ProFUM07) had at least one bacterial taxon whose level was statistically significant; three participants (ProFUM08, ProFUM05, and ProFUM06) did not; the latter two previously showed no differences in alpha-diversity values (Table S4) or JSD values (Table 2) during menstruation. Overall, changes in MSU microbial stability and composition were associated with menstruation, but participants showed individualized trends.

10.1128/mBio.00475-20.6 TABLE S4 Association between alpha-diversity values for MSU microbiota and participant-reported menstruation. Download Table S4, PDF file, 0.1 MB. Copyright © 2020 Price et al. 2020 Price et al. This content is distributed under the terms of the Creative Commons Attribution 4.0 International license.

10.1128/mBio.00475-20.7 TABLE S5 List of MSU taxa with significantly different frequencies of detection during (A) participant-reported menstruation and (B) participant-reported vaginal intercourse. Download Table S5, PDF file, 0.1 MB. Copyright © 2020 Price et al. 2020 Price et al. This content is distributed under the terms of the Creative Commons Attribution 4.0 International license.

### Sexual activity and LUT microbiota variability.

Sexual activity was reported by six participants (Table S6). Of these six participants, penetrative vaginal intercourse was reported by all at least once and was the most commonly reported form of sexual activity. For the applicable participants (ProFUM06, ProFUM07, and ProFUM08), condom use was reported for 100% of the reported instances of vaginal intercourse. One participant, ProFUM07, reported being with the same male partner for all instances of vaginal intercourse (5/5, 100%), while ProFUM08 reported different male partners for each instance of vaginal intercourse (0/3, 0%). Five of the six participants reported receiving oral sex (Table S6).

10.1128/mBio.00475-20.8 TABLE S6 Summary of participant-reported personal factors relating to sexual activity. Download Table S6, PDF file, 0.05 MB. Copyright © 2020 Price et al. 2020 Price et al. This content is distributed under the terms of the Creative Commons Attribution 4.0 International license.

Significantly higher median JSD values for the MSU microbiotas but not the periurethral microbiotas were associated with participant-reported sexual activity for three of the six applicable participants: ProFUM05, ProFUM06, and ProFUM08 (Table S7A). We next separated the responses by type of sexual activity; four of the six applicable participants (ProFUM02, ProFUM05, ProFUM07, and ProFUM08) had significant associations between MSU microbiota JSD values and vaginal intercourse, while only two participants (ProFUM02 and ProFUM08) had an association with oral sex (Table 3). However, the two participants with significant associations between MSU microbiota JSD values and oral sex were the only two participants who always coreported oral sex with vaginal intercourse, suggesting that the trend associated with sexual activity may be due to vaginal intercourse.

**TABLE 3:** Association between Jensen-Shannon divergence values for MSU microbiota and participant-reported vaginal intercourse and oral sex

Participant	Median JSD value or *P* value^*a*^					
	Penetrative vaginal intercourse			Receiving oral sex		
	Yes (*n*)	No (*n*)	*P* value	Yes (*n*)	No (*n*)	*P* value
ProFUM01	N/A	N/A		N/A	N/A	
ProFUM02	0.559 (2)	0.136 (64)	**<0.001**	0.559 (2)	0.136 (64)	**<0.001**
ProFUM03	N/A	N/A		N/A	N/A	
ProFUM04	0.057 (27)	0.063 (45)	0.373	N/A	N/A	
ProFUM05	0.200 (21)	0.108 (46)	**0.036**	0.264 (4)	0.125 (63)	0.078
ProFUM06 (16S)	0.173 (1)	0.123 (65)	0.152	0.150 (2)	0.123 (64)	0.183
ProFUM07	0.242 (4)	0.057 (65)	**<0.001**	0.163 (4)	0.057 (65)	0.094
ProFUM07 (16S)	0.057 (4)	0.054 (65)	0.150	0.083 (4)	0.054 (65)	0.117
ProFUM08	0.279 (3)	0.071 (64)	**<0.001**	0.279 (3)	0.071 (64)	**<0.001**

a Median JSD values are shown for MSU microbiota on days when the participant reported (“Yes”) or did not report (“No”) vaginal intercourse or oral sex. The Mann-Whitney U test was used for statistical analyses. *P* values of <0.05 represent statistical significance (bold). N/A (not applicable), participant did not report the personal factor. *n*, number of participants.

10.1128/mBio.00475-20.9 TABLE S7 (A) Association between JSD values for MSU and periurethral microbiota and participant-reported sexual activity. (B) Association between alpha-diversity values for MSU microbiota and participant-reported vaginal intercourse. Download Table S7, PDF file, 0.1 MB. Copyright © 2020 Price et al. 2020 Price et al. This content is distributed under the terms of the Creative Commons Attribution 4.0 International license.

To determine the nature of MSU microbiota variability associated with vaginal intercourse, we assayed for differences in microbiota composition and diversity between days with and without vaginal intercourse reported. Median alpha-diversity measures following vaginal intercourse were significantly higher for two participants (Table S7B). Four participants had at least one bacterial taxon whose level was statistically significant (Table S5B). The exceptions were participants ProFUM02 and ProFUM06; however, since these participants reported vaginal intercourse on only 2 days and 1 day, respectively, the power of analysis to detect significant changes in the taxa was low. Participants ProFUM04, ProFUM05, ProFUM07, and ProFUM08 all had significantly higher frequencies of detection of various *Streptococcus* and *Staphylococcus* species following vaginal intercourse. Participant ProFUM02 also had higher frequencies of *Streptococcus* species, but the data were not statistically significant (*P = *0.081). Table S5B also shows that some species, none of which belonged to genus *Streptococcus* or genus *Staphylococcus*, had significantly lower frequencies of detection following vaginal intercourse. These data may affect the alpha-diversity values seen in Table S7B. Overall, changes in MSU microbial composition were associated with vaginal intercourse, but unlike with menstruation, we observed similar trends both within and among participants.

### Assessing confounding factors.

We next determined if the described trends were confounded by other reported factors. Tampon and/or pad use nearly always cooccurred with menstruation, as did the daily number of bowel movements for three participants (Table S8A), while oral sex frequently cooccurred with vaginal intercourse (Table S8B).

10.1128/mBio.00475-20.10 TABLE S8 (A) Significance of reporting menstruation with potentially confounding personal factors. (B) Significance of reporting vaginal intercourse with potentially confounding personal factors. Download Table S8, PDF file, 0.1 MB. Copyright © 2020 Price et al. 2020 Price et al. This content is distributed under the terms of the Creative Commons Attribution 4.0 International license.

### Urine property dynamics.

To determine if the described trends resulted from an altered urinary environment, we assayed for relationships between personal factors and urine properties, as measured by a urine dipstick. With few exceptions, blood presence represented the only significantly altered dipstick-measured urine property during and menstruation (Table 4). No changes in any of the dipstick measures were associated with vaginal intercourse (Table 4).

**TABLE 4:** Significance of participant-reported personal factors with dipstick-measured urine property results^*c*^

Category and participant	*P* value for indicated factor (no. of coreports/total no. of reports)								
	Bilirubin (*n*)	Ketones (*n*)	sp gr (osmolarity)	Blood (*n*)	pH	Protein (*n*)	Urobilirubin (*n*)	Nitrites (*n*)	Leukocytes (*n*)
Menstruation									
ProFUM01	1.000^*a*^ (3/15)	0.164^*a*^ (1/14)	**0.020**^*b*^	**<0.001**^*a*^ **(13/16)**	**0.046**^*b*^			0.400^*a*^ (1/2)	0.181^*a*^ (3/7)
ProFUM02	0.407^*a*^ (1/24)	1.000^*a*^ (1/9)	0.647^*b*^	**0.005**^*a*^ **(4/8)**	0.965^*b*^	0.665^*a*^ (1/19)	1.000^*a*^ (0/1)	1.000^*a*^ (0/1)	
ProFUM03	0.532 (5/24)	1.000^*a*^ (1/1)	0.939^*b*^	**<0.001**^*a*^ **(17/40)**	0.648^*b*^	0.166^*a*^ (3/9)			
ProFUM04	0.722^*a*^ (3/16)		0.144^*b*^	0.705^*a*^ (3/15)	0.583^*b*^	1.000^*a*^ (0/4)		1.000^*a*^ (0/1)	0.581^*a*^ (0/6)
ProFUM05	1.000^*a*^ (3/52)	1.000^*a*^ (0/3)	0.772^*b*^	1.000^*a*^ (0/5)	0.667^*b*^	0.452^*a*^ (1/12)		1.000^*a*^ (1/2)	1.000^*a*^ (0/2)
ProFUM06	0.696^*a*^ (5/41)	1.000^*a*^ (0/1)	0.135^*b*^	**0.003**^*a*^ **(3/8)**	0.510^*b*^			1.000^*a*^ (0/1)	0.208^*a*^ (1/2)
ProFUM07	0.075^*a*^ (5/5)	1.000^*a*^ (1/2)	0.325^*b*^	**<0.001**^*a*^ **(26/29)**	0.512^*b*^	0.141^*a*^ (4/4)		1.000^*a*^ (1/1)	0.678 (9/14)
ProFUM08	0.850 (11/40)	0.318^*a*^ (2/14)	0.862^*b*^	**0.030 (10/22)**	0.856^*b*^	0.741^*a*^ (3/14)			0.320^*a*^ (2/14)

Vaginal Intercourse									
ProFUM01	N/A	N/A	N/A	N/A	N/A	N/A	N/A	N/A	N/A
ProFUM02	0.129^*a*^ (2/24)	0.016^*a*^ (2/9)	0.289^*b*^	1.000^*a*^ (0/8)	0.638^*b*^	0.484^*a*^ (1/19)	1.000^*a*^ (0/1)	1.000^*a*^ (0/1)	
ProFUM03	N/A	N/A	N/A	N/A	N/A	N/A	N/A	N/A	N/A
ProFUM04	1.000 (6/16)		0.542^*b*^	0.822 (6/15)	0.949^*b*^	1.000^*a*^ (1/4)		1.000^*a*^ (0/1)	1.000^*a*^ (2/6)
ProFUM05	**0.007 (12/52)**	0.546^*a*^ (0/3)	0.054^*b*^	0.315^*a*^ (3/5)	**0.008**^*b*^	0.395 (5/12)		0.532^*a*^ (1/2)	1.000^*a*^ (0/2)
ProFUM06	1.000^*a*^ (1/41)	**0.015**^*a*^ **(1/1)**	0.125^*b*^	1.000^*a*^ (0/8)	0.250^*b*^			1.000^*a*^ (0/1)	1.000^*a*^ (0/2)
ProFUM07	0.265^*a*^ (1/5)	1.000^*a*^ (0/2)	0.534^*b*^	1.000^*a*^ (2/29)	1.000^*b*^	1.000^*a*^ (0/4)		1.000^*a*^ (0/1)	0.575^*a*^ (0/14)
ProFUM08	1.000^*a*^ (2/40)	0.511^*a*^ (1/14)	0.473^*b*^	0.545^*a*^ (0/22)	0.953^*b*^	1.000^*a*^ (0/14)			0.108^*a*^ (2/14)

a Fisher’s exact test.

b Wilcoxon rank sum test.

c Frequency of urination, properties of urine samples, and constituent results were assayed for associations with participant-reported vaginal intercourse. Any outcome other than “negative” was considered a positive urine test result, except for specific gravity and pH data, which do not have “negative” outcomes. *P* values are shown in the table. The chi-square test was used unless otherwise indicated. *P* values of <0.05 represent statistical significance (bold). *n*, number of participants. Data corresponding to properties and constituents for a given participant that were always negative or baseline are represented by empty cells (or by “N/A”). N/A (not applicable), participant did not report vaginal intercourse. Significant *P* values that are underlined represent urine properties and constituents with “positive” results found at higher frequencies (or higher mean values) following vaginal intercourse. The numbers in parentheses in each applicable cell represent the number of times each “positive” categorical test result was coreported with vaginal intercourse in the total number of reports. Vaginal intercourse was reported as follows: for ProFUM01, 0/67 days; for ProFUM02, 2/66 days; for ProFUM03, 0/71 days; for ProFUM04, 27/72 days; for ProFUM05, 21/67 days; for ProFUM06, 1/66 days; for ProFUM07, 4/69 days; for ProFUM08, 3/67 days.

## DISCUSSION

This study showed that the LUT microbiota is both dynamic and resilient. Our findings demonstrate variability in the voided urine sample of adult women participants, revealing the dynamic nature of the LUT microbiota and providing evidence that both menstruation and sexual activity influence those dynamics. They also show that the LUT and periurethral area are distinct microbiological niches in most young women.

Using JSD, an analytic approach commonly used to measure longitudinal stability, we found that, within an individual, the periurethral microbiota is more variable than the LUT microbiota. Physiologically, this is not surprising, as the periurethral area is exposed to the genital and external environment, while the LUT is not. Thus, transitions of flora may occur more frequently in the periurethral area than in the LUT.

Because posturethral (e.g., vulva and/or vaginal) microbes are often present in MSU samples (35, 36), it was essential to include a control for specimen quality, which is why we used a periurethral swab to measure specimen collection compliance. This periurethral control allowed us to derive conclusions that exclusively relate to the LUT microbiota rather than to those of the periurethra. Since we did not collect vaginal swabs, we cannot comment on bacterial interplay between the periurethral area and vagina. As this was the first study to assess periurethral microbiota temporal dynamics, we cannot compare our findings to those of others. It is possible that periurethral microbiota variability relates to specimen collection variability. Participants received verbal and visual instructions explaining the method of collection of the periurethral specimens by swabbing 1 cm lateral to the urethral opening. However, one would expect that with repeated collection over 3 months, the participant’s technique to collect the periurethral specimen would become more standard and consistent, meaning that the JSD values would become lower (i.e., more stable) throughout the study. Qualitatively, this did not occur, suggesting that the daily variability of the periurethral microbiota is biologically meaningful and not likely due to collection inconsistencies.

Sexual activity was associated with increased variability in the MSU—and the type of sexual activity mattered. Moreover, the levels of this sex-associated variability differed within individuals. Elevated frequencies and abundances of *Streptococcus* and *Staphylococcus* species in the MSU specimens were consistent findings following vaginal intercourse. One might predict microbiota variability to be related to oral sex. Indeed, participant ProFUM04, who reported only vaginal intercourse (27/72 days), did not have significantly different JSD values for either MSU or periurethral microbiotas (Table 3). Furthermore, the idea of a relationship between variability and oral intercourse might be supported by the fact that *Streptococcus* species are commonly part of the oral flora (37). However, if one considers the other participants’ data, this relationship is less convincing. For example, participant ProFUM05 reported sexual activity 21/67 days; 17/21 days she reported vaginal intercourse only, while the other 4/21 days she reported both vaginal and oral sex. Assessment of these latter 4 days alone shows no significant association with MSU microbiota stability, while assessment of all 21 days does (Table 3). Furthermore, participant ProFUM07 reported sexual activity 6/69 days; 2/6 days she reported only vaginal intercourse, 2/6 only oral sex, and 2/6 both. Again, only assessment of the days that included vaginal intercourse (4/6) showed significant associations with MSU microbiota stability (Table 3). Altogether, these data suggest that vaginal intercourse, rather than oral sex, is associated with MSU microbiota variability.

In the three participants who were asked (i.e., ProFUM06, ProFUM07, and ProFUM08), condom use was reported 100% of the time when vaginal intercourse was reported. Therefore, it is impossible to derive a conclusion regarding the influence of condom use on the relationship between MSU microbiota variability and vaginal intercourse from these data alone. The levels of sexual partner variability differed among the applicable participants; whereas participant ProFUM07 reported the same male sexual partner for each instance of vaginal intercourse (*n* = 4), participant ProFUM08 reported different male partners (*n* = 3). To conclusively show a role for the male sexual partner in the MSU microbiota variability of the female, it would be necessary to show movement of genetically related isolates between the partners following vaginal intercourse longitudinally. This was demonstrated previously by Eren and coworkers, who observed strong correlations between unique sequence variants of Gardnerella vaginalis strains obtained from vaginal and urethral/penile samples of sexual partners (38).

The effects of vaginal intercourse on the vaginal and MSU microbiotas appear to differ. Disruptions in the stability of the vaginal microbiota have been associated with vaginal intercourse (3). This includes an increase in *Gardnerella* (3, 39–41) sometimes accompanied by a decrease in *Lactobacillus*, particularly L. iners (39) and L. crispatus (41). But this increase is not seen when condoms are used (39). The leading theory of the mechanism behind these changes is that the altered microbiota comes from the male ejaculate, which contains microbes of the male urethra. The microbiome of semen was very recently characterized and was found to contain abundances of bacteria, many of which are part of the vaginal microbiota (e.g., *Lactobacillus*, *Veillonella*, *Streptococcus*, *Porphyromonas*, *Atopobium*), including *Gardnerella* (41). Mändar et al. recently assessed 23 heterosexual couples and found that the semen microbiota, collected several days prior to intercourse, was more similar to the partner’s vaginal microbiota after vaginal intercourse (41). We did not observe increases in *Gardnerella* levels in the MSU specimens following vaginal intercourse but instead observed increases in *Streptococcus* and *Staphylococcus* levels. *Streptococcus* is a predominant taxon in the semen microbiota (41). However, we observed these trends even when condom use was reported (e.g., ProFUM07 and ProFUM08); therefore, the bacteria were not likely from the male ejaculate. Overall, the only compatible finding is that both the vaginal and MSU microbiota have decreased stability following vaginal intercourse, but the mechanisms underlying these changes are likely very different, which is likely a reflection of the unique physiology of each site.

Menstruation (and associated activities, such as the use of menstrual products) increased microbiota variability. Whereas our data revealed trends with obvious variability (i.e., JSD values), they were highly individualized. Thus, it is difficult to determine the mechanistic relationship between menstruation and MSU microbiota variability. The microbes found at higher frequencies and abundances during menstruation (e.g., *Corynebacterium*, *Staphylococcus*, *Actinomyces*) are primarily skin flora (1). One possible explanation for their presence might be exogenous introduction via feminine hygiene product use, which was reported at significantly higher frequencies during menstruation for all participants (see Table S8 in the supplemental material). However, if hygiene products introduced bacteria to the LUT, one might expect similar changes in the periurethral area, and yet we observed no change in periurethral microbiota stability with menstruation or hygiene product use. An alternative explanation might be that the LUT environment changes during menstruation, thus favoring outgrowth of different microbes. Except for blood, however, there were no significant changes in the frequency of positive urine properties and constituent results during menstruation (Table 4). The blood likely originated from the vaginal tract and thus is not directly relevant to the LUT environment, and yet this serves as an important positive control to verify the integrity of participants’ responses to the questionnaire. In addition to feminine hygiene product use, only the reported number of bowel movements was found to have a significant association with menstruation (Table S8A). However, when we assayed for associations between number of bowel movements and microbiota characteristics, we found no significant trends. Hormonal effects are also a possibility but were not investigated here.

While we relied primarily on EQUC, we showed that those results are comparable to those obtained with 16S rRNA gene sequencing. We analyzed MSU specimens from ProFUM07 by both modified EQUC and 16S rRNA gene sequencing (Fig. 3G and H, respectively). Qualitatively, the microbiota and microbiome were dominated by *Lactobacillus* (blue) except for three 1-to-2-week periods during the beginning (i.e., week 1), middle (i.e., weeks 3 and 4), and end (i.e., weeks 7 and 8) of the study period. This deviation from *Lactobacillus* dominance was shown to have occurred for the same MSU specimens by both methods. Appropriately, JSD values were elevated for both methods during these periods. In fact, the JSD values corresponding to the two methods followed almost identical patterns over time (see Fig. S2 in the supplemental material). These data showed that the two methods are complementary and validated the use of only one method to analyze the specimens of the other seven participants.

10.1128/mBio.00475-20.2 FIG S2 JSD values for MSU microbiota of participant ProFUM07 obtained using culture and sequencing. JSD values were calculated for MSU microbiota of participant ProFUM07. Microbiota data were obtained using modified EQUC (solid line) and 16S rRNA gene sequencing (dashed line). Data are plotted over time (in days). Download FIG S2, TIF file, 0.1 MB. Copyright © 2020 Price et al. 2020 Price et al. This content is distributed under the terms of the Creative Commons Attribution 4.0 International license.

We have limited our approach to assessing the relationship between personal factors and the microbiota of the subsequent day. It is difficult to discern whether it would be biologically appropriate to further expand the analyses. It is possible that some personal factors take longer than 1 day to significantly impact the microbiota. In previous studies, correlations between personal factors and UTI risk, for example, were studied across weeks, months, and even years and thus do not provide relevant information regarding the timeliness of their effects. Nonetheless, our data set represents a novel means to determine if relationships between personal factors and delayed changes to the LUT microbiota exist, which is an important direction to consider for future study.

This report shows that it is feasible to perform longitudinal urine specimen collection in premenopausal sexually active women, although our “screen” for sample concordance excluded 1/3 of the potential participants. This screening threshold may require revision with subsequent studies, as it may have biased our participant population; excluding participants on the basis of a lack of distinct microbiota between paired specimens may not actually relate to specimen collection compliance. Although others have previously suggested that the microbes of the periurethral area and MSU are distinct (42–44), rigorous and properly controlled studies assessing this distinction have yet to be performed. Our data show that these two niches are distinct in some women.

## MATERIALS AND METHODS

### ProFUM study enrollment and specimen collection.

From September 2017 to July 2018, we invited asymptomatic premenopausal female employees from the Loyola University Medical Center campus to participate in an institutional review board (IRB)-approved clinical trial (ClinicalTrials registration no. NCT03250208) entitled “Probiotics and the Female Urinary Microbiome (ProFUM) study.” The study was divided into three phases. Phases I and III flanked an experimental phase (i.e., phase II), in which the participants were randomized to take an oral probiotic or placebo. This part of the study was published previously (34). MSU, periurethral swabs, and a personal questionnaire were collected daily throughout all three phases.

Using IRB-approved invitation methods, potentially interested individuals contacted a research nurse and were screened for eligibility during an in-person visit. Inclusion criteria were female gender at birth, age over 18 years, and ability to read English and sign a consent form detailing the requirements and voluntary nature of the study. Exclusion criteria were current pregnancy, antibiotic or probiotic usage, or a plan to vacation for more than 7 days during the time of specimen collection (i.e., 3 months). Individuals who met these eligibility criteria signed a consent form and were instructed that a 3-day sample collection screening was required prior to final enrollment in the study. This is described below.

Participants were given sufficient supplies (described below) and instructed on how to collect, label, and deliver daily MSU and periurethral swab specimens to the research team. Participants were instructed on proper specimen collection through use of a standardized video detailing proper collection of an MSU specimen and a periurethral swab. Specifically, participants were instructed not to use the genital cleansing wipe provided in the urine collection kits. Participants were assigned unique study identifiers (IDs) by the research nurse and were instructed to attach labels with their study ID and date of collection to each specimen prior to delivery to a locked drop-box in an accessible room at Loyola University Medical Center.

MSU specimens were collected by voiding into a toilet to discard the initial void (i.e., approximately the first 10 ml of urine). The remaining specimen was then collected into a sterile collection cup. A portion of each urine sample was placed in a sterile manner into a BD Vacutainer Plus C&S preservative tube for culturing. The tube contained a lyophilized boric acid preservative that prevented bacterial overgrowth without causing cell death, allowing specimens to be held at room temperature for up to 72 h without altering integrity. Specimens that were collected more than 72 h prior to receipt were excluded from analyses.

Periurethral swab specimens were collected using a BD ESwab Liquid Amies collection and transport system. Use of these flocked swabs allows optimal elution of the specimen into the medium. Specimens were stored at room temperature. Due to lack of a preservative, specimens that were collected more than 48 h prior to receipt were excluded from analyses due to the possibility of bacterial overgrowth and subsequent inaccurate data. Periurethral swabs were collected by swabbing vertically top to bottom approximately 1 cm lateral to the urethral opening.

Study specimen collection was initiated with a 3-day assessment to verify proper specimen collection. Participants were withdrawn from further study participation if their MSU and periurethral specimens had similar microbial contents, as determined using the Bray-Curtis dissimilarity test. A value of 0.8 was the threshold for eligibility. Participants whose paired specimens showed scores of >0.8 for two of three collection days of the screening period continued in the study. The continuing participants were scheduled for a second one-on-one meeting with the clinical team, during which the continuing research tasks associated with the ProFUM study were described and participants completed a demographics questionnaire, which included the following parameters: age, race/ethnicity, height/weight, blood pressure, vaginal parity, birth control method, condom use, typical length of menstrual cycles, use of menstrual hygiene products, prior urogynecologic surgery, sexual activity (frequency, type, partners), dietary preferences, alcohol consumption, number of bowel movements in an average week, use of cigarettes, frequency of bathing, current medications, and other relevant data (UTI, kidney stones, urinary incontinence, and fecal incontinence).

In addition to specimen collection, participants completed a daily personal questionnaire regarding life events and behaviors of the previous 24 h. The nonvalidated questionnaire queried alcohol consumption, medications, medical events, menstruation, menstrual hygiene, bathing, swimming, sexual activity (oral, vaginal, other), and number of bowel movements. The questionnaire included two yes/no-style questions regarding urination and bowel movements for the current day (i.e., since waking up and before specimen collection). The last three participants completed a modified questionnaire that included two additional questions regarding condom use with vaginal intercourse and whether the sexual partner had changed. Completed questionnaires were given to the research team with each specimen.

### Laboratory analysis.

A modified version of EQUC protocol was conducted to identify microbiota (27). For the MSU specimens, 0.01 ml of urine was spread quantitatively onto diverse types of media (blood agar plate [BAP], Columbia nalidixic acid [CNA] agar, and CDC anaerobe 5% BAP [ABAP]) and incubated in appropriate environments at appropriate temperatures (5% CO_2_ at 35°C for 48 h or anaerobic conditions at 35°C for 48 h). Each morphologically distinct colony type was counted and isolated to prepare a pure culture for identification by matrix-assisted laser desorption ionization–time of flight mass spectrometry (MALDI-TOF MS), using a Bruker MALDI Biotyper Research (research-use-only [RUO]) system. All swab specimens underwent the same protocol using 0.01 ml of the liquid elution media after the swab was subjected to vortex mixing in the collection tube for 10 s.

Each MSU specimen was characterized using a urine dipstick. Approximately 1 ml of urine was pipetted onto a Siemens Multistix 10 SG reagent strip. The reagent strip tests for the presence and quantity (or relative quantity) of glucose, bilirubin, ketones, hemoglobin (i.e., blood), protein, urobilinogen, nitrites, and leukocytes. These strip tests also measure pH and specific gravity. Results were read and interpreted according to manufacturer’s instructions.

The remaining volumes of the MSU and swab specimen elution media after bacterial culture were aliquoted for 16S rRNA gene sequencing. The nucleic acid preservative AssayAssure (Sierra Molecular, Incline Village, NV) was added (10% relative to specimen volume) before storage at –80°C.

DNA isolation, PCR amplification, and 16S rRNA gene sequencing of urine cultures have been described previously (45). To minimize contamination, isolation of DNA was performed in a laminar flow hood. Genomic DNA was extracted from MSU and swab elution media with a Qiagen DNeasy blood and tissue kit. A 1-ml volume of urine or 0.5 ml of swab elution media was used. Peptidoglycan-degrading enzymes (mutanolysin and lysozyme) were added to ensure robust lysis of Gram-positive and Gram-negative species (46). DNA was eluted into 50 μl of buffer AE (10 mM Tris-Cl, 0.5 mM EDTA [pH 8.0]) and stored at –20°C. Hypervariable region 4 (V4) of the bacterial 16S rRNA gene was amplified via a two-step PCR protocol (27, 31). The PCR was purified and subjected to size selection using Agencourt AMPure XP-PCR magnetic beads (Beckman Coulter, Pasadena, CA). Each sample was quantified using a Qubit fluorometric system (Thermo Fisher, Waltham, MA). The samples were pooled, quantified to a standard volume, and placed in the 2-by-250-bp sequencing reagent cartridge, according to the instructions of the manufacturer (Illumina, San Diego, CA).

After sequencing, sample barcodes and sequencing primers were removed using Illumina proprietary MiSeq postsequencing software. The mothur program (v1.41.3) was used to process raw sequences following the recommended MiSeq standard operating procedure (47). Briefly, mothur produced 16S contigs by combining paired-end reads based on overlapping nucleotides in sequence reads; contigs of incorrect length for the V4 region (<290 bp, >300 bp) and/or contigs containing ambiguous bases were removed. Chimeric sequences were removed using UCHIME within the mothur package (48). Subsampling at a depth of 5,000 sequences was performed to correct for the different sequencing depths of each sample. The sequences were clustered into species-level operational taxonomic units (OTUs) with identity cutoff at 97% (49). The OTUs were classified using RDP classifier (v2.11) at the genus level (49) and BLCA (Bayesian lowest common ancestor) (50) at the species level.

### Statistical analyses.

No *a priori* power calculation was performed to determine the appropriate sample size for this exploratory study; sample size was determined by feasibility, research team availability, and budgetary constraints.

Statistical analyses of microbiota data were performed using SAS software version 9.4 (SAS, Cary, NC). Microbiota stability measures were calculated using Jensen-Shannon divergence (JSD) as described previously (2). First, we calculated a representative microbiota distribution for each participant, averaging the abundance data for the microbiota across all collection days. JSD values were then calculated by comparisons between the values representing each day’s microbiota and the average microbiota for each participant. These values were then applied to metadata, such as questionnaire results, to determine if a statistical relationship existed between microbiota stability and personal factors. Wilcoxon rank sum tests and Mann-Whitney U tests compared mean or median JSD values with participant-reported personal factors. Frequency of bacterial detection was compared to personal factors using either Pearson chi-square or Fisher’s exact tests, depending on assumption validity. Chi-square testing was used to compare categorical variables. One-way analysis of variance (ANOVA) was used to compare continuous variables. Correlations between variables were determined using the Pearson correlation test. All test results were considered significant using a *P* value of <0.05. Relative abundance graphs, alpha diversity measures, and PCoA plots were generated in RStudio.

### Data availability.

DNA sequences have been deposited in the SRA at NCBI, accession number PRJNA613018.
