# Supplementary material for: Temporal Dynamics of the Adult Female Lower Urinary Tract Microbiota
Source: mBio. 2020 Apr 21;11(2):e00475-20. doi: 10.1128/mBio.00475-20 (PMC7175091; doi:10.1128/mBio.00475-20)
Supplement: TABLE S1 [file mBio.00475-20-st001.pdf]

Supplemental Table 1. Bray-Curtis Dissimilarity Values for Paired Specimens during the Screening Phase.

Screen ID	Day 1	Day 2	Day 3	Result	Screen ID	Day 1	Day 2	Day 3	Result
**SCREEN01**	*0.94*	*0.86*	*0.80*	PASS	**SCREEN07**	*0.57*	*0.92*	*0.89*	PASS
**SCREEN02**	*0.01*	*0.22*	*0.38*	X	**SCREEN08**	*0.35*	*0.94*	*0.81*	PASS
**SCREEN03**	*0.96*	*0.94*	*0.82*	PASS	**SCREEN09**	*0.33*	*0.30*	*0.70*	X
**SCREEN04**	*0.28*	*0.44*	*0.41*	X	**SCREEN10**	*0.44*	*0.46*	*0.28*	X
**SCREEN05**	*0.89*	*0.99*	*0.51*	PASS	**SCREEN11**	*0.91*	*0.83*	*0.79*	PASS
**SCREEN06**	*0.87*	*0.67*	*0.85*	PASS	**SCREEN12**	*0.93*	*0.96*	*0.97*	PASS

"PASS" indicates that the participant was eligible for the ProFUM study; X indicates that the participant was not eligible for the ProFUM study; Bray-Curtis Dissimilarity values < 0.80 are highlighted in grey.
