# Supplementary material for: Temporal Dynamics of the Adult Female Lower Urinary Tract Microbiota
Source: mBio. 2020 Apr 21;11(2):e00475-20. doi: 10.1128/mBio.00475-20 (PMC7175091; doi:10.1128/mBio.00475-20)
Supplement: TABLE S2 [file mBio.00475-20-st002.pdf]

Supplemental Table 2. Overview of Bray-Curtis Dissimilarity Values Comparing MSU and Peri-urethral Microbiota of the ProFUM Participants based on EQUC data.

	Median	Interquartile Range (IQR)
**ProFUM01**	*0.98*	*0.97-1.00*
**ProFUM02**	*0.91*	*0.73-0.99*
**ProFUM03**	*0.80*	*0.69-0.84*
**ProFUM04**	*0.86*	*0.66-0.96*
**ProFUM05**	*0.86*	*0.68-0.99*
**ProFUM06**	-	-
**ProFUM07**	*0.85*	*0.62-0.96*
**ProFUM08**	*0.80*	*0.52-0.92*

Specimens of participant ProFUM06 were not cultured.
