# Supplementary material for: Temporal Dynamics of the Adult Female Lower Urinary Tract Microbiota
Source: mBio. 2020 Apr 21;11(2):e00475-20. doi: 10.1128/mBio.00475-20 (PMC7175091; doi:10.1128/mBio.00475-20)
Supplement: TABLE S3 [file mBio.00475-20-st003.pdf]

Supplemental Table 3. Qualitative Description of the Microbial Temporal Dynamics of MSU Specimens of the ProFUM Participants shown in Figure 3.

Participants	Figure 3 Panel	Brief Demographic Description	Description of Microbial Dynamics of MSU Specimens
**ProFUM01**	**A**	• White/Caucasian<br>• Regular menses<br>• Condom use as only form of birth control	Microbiota alternated between *Lactobacillus* (blue) and *Gardnerella* (red) dominance with short periods of elevated *Streptococcus* (green) and *Corynebacterium* (orange) abundance. The *Lactobacillus* were exclusively identified as *L. crispatus*, while the *Gardnerella* were a mixture of *G. vaginalis* and *G. species.*
**ProFUM02**	**B**	• White/Caucasian<br>• Irregular menses<br>• IUD use for birth control	Microbiota were primarily *Lactobacillus*-dominant. The *Lactobacillus* species were a mix of *L. jensenii* and *L. crispatus*, with generally higher abundances of *L. crispatus*. The primary exception to the *Lactobacillus* dominance was a roughly two-week period during the first month of specimen collection, where specimens were dominated by *G. vaginalis*, and *Corynebacterium tuberculostearicum*.
**ProFUM03**	**C**	• Asian<br>• Regular menses<br>• IUD use for birth control<br>• One or more previous vaginal deliveries	ProFUM03 was the only participant without *Lactobacillus* identified as part of her longitudinal MSU microbiota or microbiome. Instead, the microbiota were a somewhat equal mix of *Streptococcus agalactiae* (i.e., Group B *Streptococcus*), *Staphylococcus epidermidis* (light blue), and a variety of *Corynebacterium* species. During three separate, roughly one-week periods at the beginning, middle, and end of specimen collection, there was an appearance of *Actinomyces neuii* (brown).
**ProFUM04**	**D**	• White/Caucasian<br>• Regular menses<br>• Daily oral birth control pill	Microbiota were almost exclusively *Lactobacillus*-dominant comprised of an equal abundance of *L. jensenii* and *L. iners*. Most specimens also had low abundances (<50%) of a variety of other microorganisms, including anaerobes such as *Finegoldia magna* (yellow), and *Anaerococcus* species (bright red), facultative anaerobes like *Actinomyces neuii* and *Corynebacterium* species, and aerobes such as *Staphylococcus*.
**ProFUM05**	**E**	• White/Caucasian<br>• "No menses" (reported scant menses during study)<br>• IUD use for birth	Similar trends to ProFUM01. Microbiota alternated between *Lactobacillus* and *Gardnerella*-dominance. The *Lactobacillus* was a mix of *L. jensenii* and *L. crispatus*, with higher abundances of *L. jensenii*, while the *Gardnerella* was exclusively *G. vaginalis*. There were periodic

		control	instances of the appearance and/or dominance of *Streptococcus*, which was primarily identified as *S. mitis*.
**ProFUM06 (16S)**	**F**	• White/Caucasian<br>• Regular menses<br>• Condom use as only form of birth control	Panel shows the relative abundance of the 20 most abundant genera. The remaining taxa were grouped into the "other" category (grey), which represents <20% relative abundance for all MSU specimens. *Lactobacillus* was the most abundant genus for most specimens. Levels of *Corynebacterium* and *Prevotella* (dark green) fluctuated over time. Low abundances of various anaerobic genera [e.g., *Anaerococcus*, *Finegoldia*, *Dialister* (bright blue), *Peptoniphilus* (light pink), *Veillonella* (dark pink)] were found in most specimens.
**ProFUM07**	**G**	• Asian<br>• Irregular menses<br>• IUD use for birth control	Similar to ProFUM03, three separate one-to-two week-long periods at the beginning, middle, and end of specimen collection were not dominated by the typical pattern of microorganisms (i.e., *Lactobacillus*). During these periods, a mix of *Corynebacterium* species, *Actinomyces radingae*, and various *Staphylococcus* and *Streptococcus* species were observed. There were periodic instances of the appearance and/or dominance of *Streptococcus*, which included a variety of species.
**ProFUM07 (16S)**	**H**		Panel shows the relative abundance of the 20 most abundant genera. The remaining taxa were grouped into the "other" category (grey), which represents <20% relative abundance for all MSU specimens. *Lactobacillus* was the most abundant genus for most specimens. Similar to ProFUM03, three separate one-to-two week-long periods at the beginning, middle, and end of specimen collection were not dominated by the typical pattern of microorganisms (i.e., *Lactobacillus*). During these periods, elevated abundances of *Corynebacterium* and *Streptococcus* and various anaerobes including *Prevotella*, *Peptoniphilus*, *Anaerococcus*, and *Dialister* were observed.
**ProFUM08**	**I**	• Black/African American<br>• Regular menses<br>• IUD use and daily oral birth control pill	Microbiota were almost exclusively *Lactobacillus*-dominant comprised of an equal abundance of *L. jensenii* and *L. crispatus*. Most specimens also had low abundances (<20%) of *G. vaginalis*. The exceptions were three separate periods within the first month of specimen collection, where the relative abundance of

			Lactobacillus and *Gardnerella* decreased while various counts of *Streptococcus* and *Staphylococcus* species appeared.
