# Supplementary figures and images for: Temporal Dynamics of the Adult Female Lower Urinary Tract Microbiota
Source: mBio. 2020 Apr 21;11(2):e00475-20. doi: 10.1128/mBio.00475-20 (PMC7175091; doi:10.1128/mBio.00475-20)

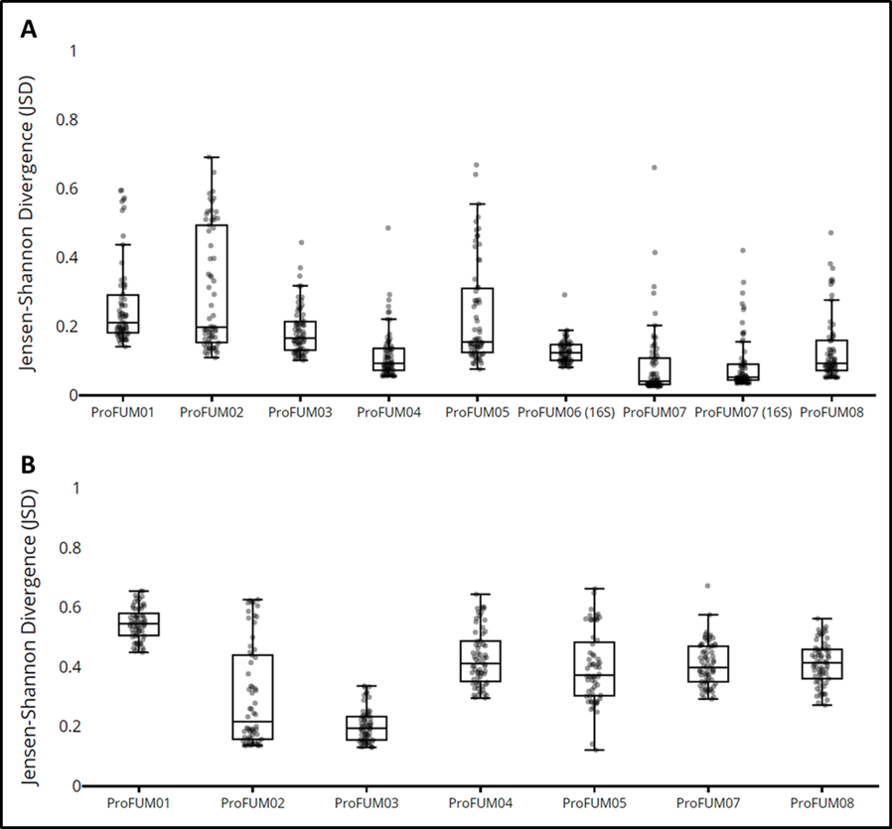

Supplement: FIG S1 [file mBio.00475-20-sf001.tif]

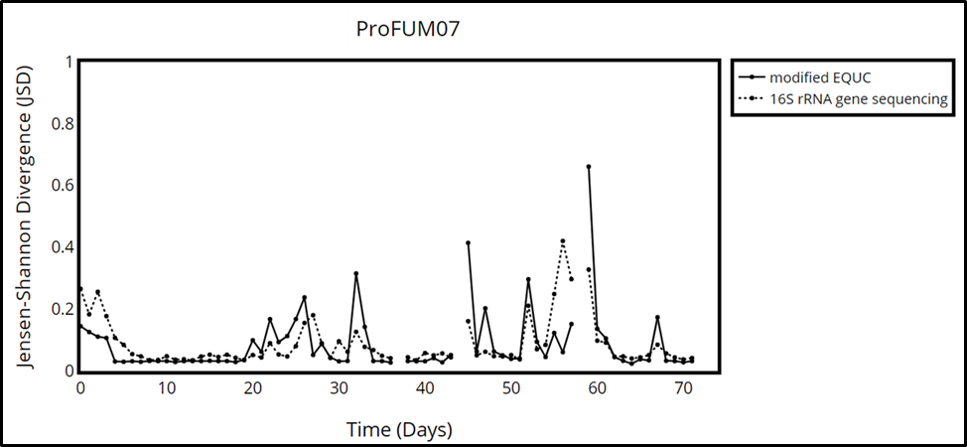

Supplement: FIG S2 [file mBio.00475-20-sf002.tif]
