# Supplementary material for: Temporal Dynamics of the Adult Female Lower Urinary Tract Microbiota
Source: mBio. 2020 Apr 21;11(2):e00475-20. doi: 10.1128/mBio.00475-20 (PMC7175091; doi:10.1128/mBio.00475-20)
Supplement: TABLE S4 [file mBio.00475-20-st004.pdf]

Supplemental Table 4. Association between Alpha-Diversity Values for MSU Microbiota and Participant-reported Menstruation.

Menstruation						
**Participants**	**MSU Microbiota (Median Shannon Diversity Values)**			**MSU Microbiota (Median Simpson's Diversity Values)**		
	**"Yes"**	**"No"**	***p*-value**	**"Yes"**	**"No"**	***p*-value**
**ProFUM01**	0.970	0.675	*0.035*	0.500	0.429	*0.040*
**ProFUM02**	1.153	0.547	*0.012*	0.557	0.307	*0.021*
**ProFUM03**	1.418	1.186	*<0.001*	0.714	0.642	*<0.001*
**ProFUM04**	1.072	0.757	*0.026*	0.584	0.489	*0.064*
**ProFUM05**	0.624	0.917	*0.168*	0.338	0.509	*0.187*
**ProFUM06 (16S)**	2.838	2.655	*0.148*	0.841	0.796	*0.057*
**ProFUM07**	0.675	0.019	*<0.001*	0.362	0.005	*<0.001*
**ProFUM07 (16S)**	1.444	1.010	*<0.001*	0.552	0.447	*0.008*
**ProFUM08**	0.883	0.778	*0.037*	0.526	0.486	*0.049*

Median Shannon Diversity (*left*) and Simpson's Diversity (*right*) values shown for MSU microbiota on days when the participant reported ("Yes") or did not report ("No") menstruation. Mann-Whitney U test used to determine significance. p-value < 0.05 is significant (green). Menstruation reported: ProFUM01 (15/67 days), ProFUM02 (7/66 days), ProFUM03 (18/71 days), ProFUM04 (12/72 days), ProFUM05 (3/67 days), ProFUM06 (7/66 days), ProFUM07 (41/69 days), ProFUM08 (19/67 days).
