# Supplementary material for: Temporal Dynamics of the Adult Female Lower Urinary Tract Microbiota
Source: mBio. 2020 Apr 21;11(2):e00475-20. doi: 10.1128/mBio.00475-20 (PMC7175091; doi:10.1128/mBio.00475-20)
Supplement: TABLE S5 [file mBio.00475-20-st005.pdf]

Supplemental Table 5. List of MSU Taxa with Significantly different Frequencies of Detection during:

(A) Participant-reported Menstruation

Participants	Significant Taxa	Frequency of Detection during Menstruation		
**ProFUM01**	**Species**	**“Yes” (*n*=15)**	**“No” (*n*=52)**	***p*-value**
	Corynebacterium lipophile group F1	6 (40%)	4 (8%)	*0.006*[a]
	Corynebacterium tuberculostearicum	7 (47%)	3 (6%)	*<0.001*[a]
	Staphylococcus epidermidis	13 (87%)	30 (58%)	*0.038*[a]
	Streptococcus agalactiae	12 (80%)	17 (33%)	*0.002*[a]
**ProFUM02**	**Species**	**“Yes” (*n*=7)**	**“No” (*n*=59)**	***p*-value**
	Actinomyces neuii	4 (57%)	5 (8%)	*0.005*[a]
	Alloscardovia omnicolens	2 (29%)	0 (0%)	*0.010*[a]
	Corynebacterium amycolatum	2 (29%)	0 (0%)	*0.010*[a]
	Corynebacterium tuberculostearicum	5 (71%)	11 (19%)	*0.007*[a]
	Gardnerella vaginalis	4 (57%)	10 (17%)	*0.032*[a]
	Staphylococcus epidermidis	7 (100%)	17 (29%)	*<0.001*[a]
	Streptococcus anginosus	6 (86%)	9 (15%)	*<0.001*[a]
**ProFUM03**	**Species**	**“Yes” (*n*=18)**	**“No” (*n*=53)**	***p*-value**
	Actinomyces neuii	17 (89%)	23 (43%)	*<0.001*[a]
	Corynebacterium amycolatum	16 (84%)	28 (53%)	*0.027*[a]
	Finegoldia magna	7 (37%)	3 (6%)	*0.002*[a]
**ProFUM04**	**Species**	**“Yes” (*n*=12)**	**“No” (*n*=60)**	***p*-value**
	Corynebacterium glucuronolyticum	3 (25%)	2 (3%)	*0.030*[a]
**ProFUM05**	**Species**	**“Yes” (*n*=3)**	**“No” (*n*=64)**	***p*-value**
	None	-	-	-
**ProFUM06 (16S)**	**Genera**	**“Yes” (*n*=7)**	**“No” (*n*=59)**	***p*-value**
	None	-	-	-
**ProFUM07**	**Species**	**“Yes” (*n*=41)**	**“No” (*n*=28)**	***p*-value**
	Actinomyces radingae	14 (34%)	1 (4%)	*0.003*[a]
	Corynebacterium amycolatum	19 (46%)	0 (0%)	*<0.001*[a]
	Corynebacterium aurimucosum	17 (41%)	2 (7%)	*0.002*[a]
	Corynebacterium tuberculostearicum	13 (32%)	2 (7%)	*0.018*[a]
	Finegoldia magna	8 (20%)	0 (0%)	*0.018*[a]
	Staphylococcus epidermidis	35 (85%)	9 (32%)	*<0.001*
	Staphylococcus hominis	12 (29%)	2 (7%)	*0.033*[a]
	Staphylococcus pettenkoferi	12 (29%)	2 (7%)	*0.033*[a]
**ProFUM07(16S)**	**Genera**	**“Yes” (*n*=43)**	**“No” (*n*=29)**	***p*-value**
	Finegoldia	7 (16%)	0 (0%)	*0.037*[a]
**ProFUM08**	**Species**	**“Yes” (*n*=19)**	**“No” (*n*=48)**	***p*-value**
	None	-	-	-

MSU microbiota listed had significantly different ($p<0.05$) frequencies of detection between menstruation and non-menstruation. Green cells indicate higher frequency. Chi-square used unless otherwise indicated. a: Fisher’s exact test

(B) Participant-reported Vaginal Intercourse

Participants	Significant Taxa	Frequency of Detection following Vaginal Intercourse		
**ProFUM02**	**Species**	**"Yes" (*n*=2)**	**"No" (*n*=64)**	***p*-value**
	None	-	-	-
**ProFUM04**	**Species**	**"Yes" (*n*=27)**	**"No" (*n*=45)**	***p*-value**
	Staphylococcus epidermidis	22 (81%)	26 (58%)	*0.039*
**ProFUM05**	**Species**	**"Yes" (*n*=21)**	**"No" (*n*=46)**	***p*-value**
	Actinomyces neuii	4 (17%)	24 (52%)	*0.016*[a]
	Lactobacillus crispatus	9 (43%)	34 (74%)	*0.014*
	Streptococcus agalactiae	5 (24%)	2 (4%)	*0.027*[a]
**ProFUM06 (16S)**	**Genera**	**"Yes" (*n*=1)**	**"No" (*n*=65)**	***p*-value**
**ProFUM07**	**Species**	**"Yes" (*n*=4)**	**"No" (*n*=65)**	***p*-value**
	Staphylococcus hominis	4 (100%)	10 (15%)	*0.001*[a]
	Streptococcus mitis	3 (75%)	0 (0%)	*<0.001*[a]
	Streptococcus salivarius	2 (50%)	0 (0%)	*0.003*[a]
**ProFUM07 (16S)**	**Genera**	**"Yes" (*n*=4)**	**"No" (*n*=65)**	***p*-value**
	Campylobacter	1 (25%)	51 (78%)	*0.044*[a]
**ProFUM08**	**Species**	**"Yes" (*n*=3)**	**"No" (*n*=64)**	***p*-value**
	Corynebacterium tuberculostearicum	2 (67%)	7 (11%)	*0.045*[a]
	Neisseria macacae	1 (33%)	0 (0%)	*0.045*[a]
	Staphylococcus hominis	3 (100%)	8 (13%)	*0.003*[a]
	Staphylococcus lugdunensis	2 (67%)	2 (3%)	*0.008*[a]
	Streptococcus gordonii	1 (33%)	0 (0%)	*0.045*[a]
	Streptococcus mitis	3 (100%)	2 (3%)	*<0.001*[a]
	Streptococcus parasanguinis	2 (67%)	0 (0%)	*0.001*[a]
	Streptococcus salivarius	2 (67%)	0 (0%)	*0.001*[a]
	Streptococcus vestibularis	1 (33%)	0 (0%)	*0.045*[a]

MSU microbiota listed, had significantly different ($p<0.05$) frequencies of detection between vaginal intercourse and no vaginal intercourse. Green cells indicate higher frequency. Chi-square used unless otherwise indicated.
a: Fisher's exact test
