# Supplementary material for: Temporal Dynamics of the Adult Female Lower Urinary Tract Microbiota
Source: mBio. 2020 Apr 21;11(2):e00475-20. doi: 10.1128/mBio.00475-20 (PMC7175091; doi:10.1128/mBio.00475-20)
Supplement: TABLE S6 [file mBio.00475-20-st006.pdf]

**Supplemental Table 6. Summary of Participant-reported Personal Factors Relating to Sexual Activity.**

| Participants | Participant-reported Personal Factors Relating to Sexual Activity | | | | | |
|---|---|---|---|---|---|---|
| | Sexual Activity | Penetrative Vaginal Intercourse | Condom Use | Same Partner – "Yes" | Received Oral Sex | "Other" Sexual Activity |
| **ProFUM01** | 0 | N/A | - | - | N/A | N/A |
| **ProFUM02** | 3 | 2 (67%) | - | - | 2 (67%) | 1 (33%) |
| **ProFUM03** | 0 | N/A | - | - | N/A | N/A |
| **ProFUM04** | 29 | 29 (100%) | - | - | 0 (0%) | 0 (0%) |
| **ProFUM05** | 22 | 22 (100%) | - | - | 4 (18%) | 0 (0%) |
| **ProFUM06** | 4 | 2 (50%) | 2 (100%) | 1 (50%) | 3 (75%) | 1 (25%) |
| **ProFUM07** | 15 | 5 (33%) | 5 (100%) | 5 (100%) | 5 (33%) | 15 (100%) |
| **ProFUM08** | 3 | 3 (100%) | 3 (100%) | 0 (0%) | 3 (100%) | 0 (0%) |

N/A (Not applicable) refers to participants who did not report the personal factor (i.e. participants ProFUM01 and ProFUM03). Condom use and new partner were added to the personal questionnaire following the completion of participant ProFUM05. Percentage indicates proportion of sexual encounters that included the corresponding factor.
