# Supplementary material for: Temporal Dynamics of the Adult Female Lower Urinary Tract Microbiota
Source: mBio. 2020 Apr 21;11(2):e00475-20. doi: 10.1128/mBio.00475-20 (PMC7175091; doi:10.1128/mBio.00475-20)
Supplement: TABLE S7 [file mBio.00475-20-st007.pdf]

Supplemental Table 7A. Association between JSD Values for MSU and Peri-urethral Microbiota and Participant-reported Sexual Activity.

Sexual Activity						
Participants	MSU Microbiota (Median JSD Values)			Peri-urethral Microbiota (Median JSD Values)		
	"Yes" (*n*)	"No" (*n*)	*p*-value	"Yes" (*n*)	"No" (*n*)	*p*-value
**ProFUM01**	N/A	N/A	-	N/A	N/A	-
**ProFUM02**	0.549 (3)	0.139 (63)	*0.207*	0.492 (3)	0.172 (59)	*0.192*
**ProFUM03**	N/A	N/A	-	N/A	N/A	-
**ProFUM04**	0.057 (27)	0.063 (45)	*0.373*	0.303 (25)	0.256 (41)	*0.162*
**ProFUM05**	0.200 (21)	0.108 (46)	*0.036*	0.267 (20)	0.249 (40)	*0.441*
**ProFUM06 (16S)**	0.172 (3)	0.122 (63)	*0.042*	-	-	-
**ProFUM07**	0.057 (13)	0.057 (56)	*0.707*	0.215 (13)	0.272 (56)	*0.140*
**ProFUM07 (16S)**	0.063 (13)	0.053 (56)	*0.136*	-	-	-
**ProFUM08**	0.279 (3)	0.071 (64)	*<0.001*	0.198 (3)	0.279 (63)	*0.080*

Median Jensen-Shannon Divergence (JSD) values shown for MSU (*left*) and peri-urethral (*right*) microbiota on days when the participant reported ("Yes") or did not report ("No") sexual activity. Mann-Whitney U test used to determine significance. *p*-value < 0.05 is significant (green). N/A (Not applicable) refers to participants who did not report the personal factor.

Supplemental Table 7B. Association between Alpha-Diversity Values for MSU Microbiota and Participant-reported Vaginal Intercourse.

Vaginal Intercourse						
Participants	MSU Microbiota (Median Shannon Diversity Values)			MSU Microbiota (Median Simpson's Diversity Values)		
	"Yes"	"No"	*p*-value	"Yes"	"No"	*p*-value
**ProFUM01**	N/A	N/A	-	N/A	N/A	-
**ProFUM02**	1.247	0.567	*0.242*	0.642	0.318	*0.303*
**ProFUM03**	N/A	N/A	-	N/A	N/A	-
**ProFUM04**	0.748	0.810	*0.532*	0.496	0.488	*0.817*
**ProFUM05**	0.757	0.925	*0.163*	0.450	0.529	*0.223*
**ProFUM06 (16S)**	1.809	2.675	*0.212*	0.682	0.800	*0.333*
**ProFUM07**	1.643	0.249	*<0.001*	0.753	0.123	*<0.001*
**ProFUM07 (16S)**	0.909	1.264	*0.083*	0.379	0.508	*0.144*
**ProFUM08**	1.895	0.804	*<0.001*	0.820	0.488	*<0.001*

Median Shannon Diversity (*left*) and Simpson's Diversity (*right*) values shown for MSU microbiota on days when the participant reported ("Yes") or did not report ("No") vaginal intercourse. Mann-Whitney U test used to determine significance. *p*-value < 0.05

is significant (green). N/A (Not applicable) refers to participants who did not report the personal factor. Vaginal intercourse reported: ProFUM01 (0/67 days), ProFUM02 (2/66 days), ProFUM03 (0/71 days), ProFUM04 (27/72 days), ProFUM05 (21/67 days), ProFUM06 (1/66 days), ProFUM07 (4/69 days), ProFUM08 (3/67 days).
