# Supplementary material for: Temporal Dynamics of the Adult Female Lower Urinary Tract Microbiota
Source: mBio. 2020 Apr 21;11(2):e00475-20. doi: 10.1128/mBio.00475-20 (PMC7175091; doi:10.1128/mBio.00475-20)
Supplement: TABLE S8 [file mBio.00475-20-st008.pdf]

Supplemental Table 8A. Significance of Reporting Menstruation with Potentially Confounding Personal Factors.

	Significance of Reporting Menstruation with Potentially Confounding Personal Factors								
	**Probiotic/ Placebo (*n*)**	**Alcohol Use (*n*)**	**Tampon Use (*n*)**	**Pad Use (*n*)**	**Vaginal Sex (*n*)**	**Oral Sex (*n*)**	**Bathing (*n*)**	**Illness (*n*)**	**# Bowel Movements**
**ProFUM01**	*0.532*<br>(10/40)	*1.000*[a]<br>(0/1)	*<0.001*[a]<br>(15/15)	*<0.001*[a]<br>(14/14)	-	-	*1.000*[a]<br>(15/66)	*1.000*[a]<br>(0/3)	*0.918*[b]
**ProFUM02**	*<0.001*[a]<br>(0/39)	*0.617*[a]<br>(2/13)	*<0.001*[a]<br>(6/6)	*0.106*[a]<br>(1/1)	*1.000*[a]<br>(0/2)	*1.000*[a]<br>(0/1)	*0.442*[a]<br>(2/30)	*1.000*[a]<br>(0/2)	*0.623*[b]
**ProFUM03**	*0.105*<br>(6/37)	-	-	*<0.001*[a]<br>(18/18)	-	-	*0.789*<br>(9/36)	*1.000*[a]<br>(3/11)	*0.136*[b]
**ProFUM04**	*0.139*<br>(4/38)	*0.671*<br>(6/32)	*<0.001*[a]<br>(12/12)	-	*0.025*[a]<br>(1/27)	-	*0.047*[a]<br>(11/47)	-	*0.026*[b]
**ProFUM05**	*1.000*[a]<br>(2/34)	*1.000*[a]<br>(1/27)	-	-	*0.547*[a]<br>(0/21)	*1.000*[a]<br>(0/4)	*1.000*[a]<br>(3/63)	*1.000*[a]<br>(0/4)	*0.977*[b]
**ProFUM06**	*1.000*[a]<br>(4/39)	*0.426*[a]<br>(5/32)	-	-	*1.000*[a]<br>(0/1)	*1.000*[a]<br>(0/2)	*0.584*[a]<br>(7/54)	-	*0.032*[b]
**ProFUM07**	*<0.001*<br>(29/37)	*1.000*[a]<br>(1/1)	-	*<0.001*[a]<br>(39/40)	*0.642*[a]<br>(3/4)	*1.000*[a]<br>(2/4)	*1.000*[a]<br>(41/69)	*0.058*[a]<br>(0/3)	*0.057*[b]
**ProFUM08**	*0.081*<br>(7/36)	*0.411*<br>(7/30)	*<0.001*[a]<br>(6/6)	-	*1.000*[a]<br>(1/3)	*1.000*[a]<br>(1/3)	*0.850*<br>(8/27)	*1.000*[a]<br>(0/1)	*0.036*[b]

Frequency of participant-reported personal factors was assessed for an association with participant-reported menstruation. *p*-values are shown in the table. Chi-square test used unless otherwise indicated. *p*-value < 0.05 is significant (green). Cells in gray are interpreted as personal factors that were not reported by the corresponding participant. Significant *p*-values that are underlined represent personal factors reported at higher frequencies (or means) during menstruation. Number of times each personal factor was co-reported with menstruation out of total is indicated in parentheses in each cell. Menstruation reported: ProFUM01 (15/67 days), ProFUM02 (7/66 days), ProFUM03 (18/71 days), ProFUM04 (12/72 days), ProFUM05 (3/67 days), ProFUM06 (7/66 days), ProFUM07 (41/69 days), ProFUM08 (19/67 days).

a: Fisher's exact test

b: Wilcoxon rank sum test

Supplemental Table 8B. Significance of Reporting Vaginal Intercourse with Potentially Confounding Personal Factors.

	Significance of Reporting Vaginal Intercourse with Potentially Confounding Personal Factors								
	**Probiotic/ Placebo (*n*)**	**Alcohol Use (*n*)**	**Menstruation (*n*)**	**Tampon Use (*n*)**	**Pad Use (*n*)**	**Oral Sex (*n*)**	**Bathing (*n*)**	**Illness (*n*)**	**# Bowel Movements**
**ProFUM01**	N/A	N/A	N/A	N/A	N/A	N/A	N/A	N/A	N/A
**ProFUM02**	*1.000*[a] (1/39)	*<u>0.036</u>*[a] (2/13)	*1.000*[a] (0/7)	*1.000*[a] (0/6)	*1.000*[a] (0/1)	*<u>0.030</u>*[a] (1/1)	*0.203*[a] (2/30)	*1.000*[a] (0/2)	*0.215*[b]
**ProFUM03**	N/A	N/A	N/A	N/A	N/A	N/A	N/A	N/A	N/A
**ProFUM04**	*0.903* (14/38)	*0.142* (9/32)	*0.025*[a] (1/12)	*0.025*[a] (1/12)	-	-	*0.749* (17/47)	-	*0.673*[b]
**ProFUM05**	*0.479* (12/34)	*0.409* (10/27)	*0.546*[a] (0/3)	-	-	*<u>0.008</u>*[a] (4/4)	*1.000*[a] (20/63)	*0.584*[a] (2/4)	*0.473*[b]
**ProFUM06**	*0.418*[a] (0/39)	*0.478*[a] (1/32)	*1.000*[a] (0/7)	-	-	*<u>0.030</u>*[a] (1/2)	*1.000*[a] (1/54)	-	*1.000*[b]
**ProFUM07**	*0.618*[a] (3/37)	*1.000*[a] (0/4)	*0.642*[a] (3/41)	-	*0.634*[a] (3/40)	*<u>0.015</u>*[a] (2/4)	*1.000*[a] (4/69)	*1.000*[a] (0/3)	*0.399*[b]
**ProFUM08**	*1.000*[a] (2/36)	*0.583*[a] (2/30)	*1.000*[a] (1/19)	*0.249*[a] (1/6)	-	*<u><0.001</u>*[a] (3/3)	*0.226*[a] (2/27)	*1.000*[a] (0/1)	*1.000*[b]

Frequency of participant-reported personal factors was assessed for an association with participant-reported vaginal intercourse. *p*-values are shown in the table. Chi-square test used unless otherwise indicated. *p*-value < 0.05 is significant (green). Cells in gray are interpreted as personal factors that were not reported by the corresponding participant. N/A (Not applicable) refers to participants who did not report vaginal intercourse. Significant *p*-values that are underlined represent personal factors reported at higher frequencies (or means) following vaginal intercourse. Number of times each personal factor was co-reported with vaginal intercourse out of total is indicated in parentheses in each cell. Vaginal intercourse reported: ProFUM01 (0/67 days), ProFUM02 (2/66 days), ProFUM03 (0/71 days), ProFUM04 (27/72 days), ProFUM05 (21/67 days), ProFUM06 (1/66 days), ProFUM07 (4/69 days), ProFUM08 (3/67 days).

a: Fisher's exact test

b: Wilcoxon rank sum test
